# Salvianolic Acid B Attenuates Ferroptosis in Acute Kidney Injury by Targeting PRDX5

**DOI:** 10.1096/fj.202500258RR

**Published:** 2025-07-14

**Authors:** Yan Tao, Shengjun Fu, Jianzhong Lu, Beitang Fu, Shanhui Liu, Lanlan Li

**Affiliations:** ^1^ Insititue of Urology, Clinical Research Center for Urology in Gansu Province, the Second Hospital & Clinical Medical School Lanzhou University Lanzhou Gansu China; ^2^ The Fifth Affiliated Hospital of Xinjiang Medical University Ürümqi Xinjiang China

**Keywords:** acute kidney injury, ferroptosis, lipid peroxidation, PRDX5, salvianolic acid B

## Abstract

Acute kidney injury (AKI) is a common side effect of the chemotherapy agent cisplatin, and ferroptosis serves as the primary mechanism underlying cell death in renal tubular epithelium in such cases. Salvianolic acid B (SAB), a compound derived from *Salvia miltiorrhiza* , has demonstrated promising anti‐inflammatory and antioxidant properties. However, its impact on ferroptosis in the context of AKI remains to be fully explored. In this study, we utilized cisplatin‐induced and folic acid‐induced AKI models to investigate the protective mechanisms of SAB on renal tissue and tubular epithelial cell injury. The impact of SAB on renal cell ferroptosis was thoroughly examined and confirmed in both AKI models. To predict the potential mechanism through which SAB regulates ferroptosis, we employed an online target prediction database and subsequently verified the specific target proteins involved. Furthermore, we used drug affinity responsive target stability (DARTS), cellular thermal shift assay (CETSA) and molecular docking techniques to assess the binding capacity of SAB to the target protein. Our results reveal that SAB alleviated cisplatin‐ and folic acid‐induced renal dysfunction in vivo and improved cisplatin‐induced HK‐2 cell injury. Mechanistically, SAB targeted and bound to PRDX5, enhancing its redox activity, which in turn potentiated the inhibitory effect of SLC7A11 and GPX4 on cisplatin‐induced ferroptosis. Silencing PRDX5 in HK‐2 cells could partially abrogate the protective effect of SAB. These results provide strong evidence for the potential of SAB in the treatment of AKI.

## Graphical abstract

Mechanistic diagram of SAB‐mediated alleviation of AKI through the regulation of PRDX5‐mediated ferroptosis in renal tubular epithelial cells. SAB alleviates cisplatin‐ and FA‐induced AKI by inhibiting ferroptosis via PRDX5‐mediated upregulation of SLC7A11, GPX4, and FSP1. SAB binds to PRDX5, enhancing its expression, which suppresses ferroptosis and inflammation, ultimately protecting against kidney injury.

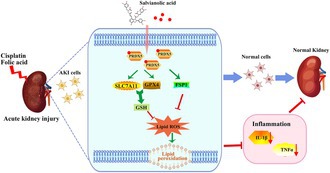

**Abbreviations**
AKI: acute kidney injury
ALT: Alanine aminotransferase
ASCL4: acyl‐CoA synthetase long‐chain family member 4
AST: Aspartate aminotransferase
BLM: bleomycin
BUN: urea nitrogen
CETSA: cellular thermal shift assay
CIS: Cisplatin
COX2: Cyclooxygenase 2
DARTS: drug affinity responsive target stability
DFO: deferoxamine
FA: Folic acid
fer‐1: ferrostatin‐1
FSP1: ferroptosis suppressor protein‐1
GPX4: glutathione peroxidase 4
GSH: glutathione
HE: Hematoxylin‐–eosin staining
HMOX1: heme oxygenase 1
IF: Immunofluorescence
IHC: Immunohistochemical
KIM‐1: Kidney injury molecule 1
Lip‐1: liproxstatin‐1.
LPS: lipopolysaccharide
MDA: malondialdehyde
NGAL: neutrophil gelatinase associated lipocalin
PCD: programmed cell death
PRDX5: Peroxiredoxin 5
qRT–PCR: quantitative real‐time PCR
ROS: reactive oxygen species
SAB: Salvianolic acid B
SLC7A11: solute carrier family 7 member 11

## 1 Introduction

The kidney is an organ with the highest metabolic rates, mainly driven by renal tubular epithelial cells. It plays a vital role in maintaining energy homeostasis, and abnormal energy metabolism can lead to cell death and various kidney diseases [1]. Acute kidney injury (AKI) is a potentially life‐threatening condition characterized by decreased urine output, a rapid increase in serum creatinine, or both, resulting in renal dysfunction. Various factors, such as sepsis, ischemia–reperfusion injury, and nephrotoxic medications, can cause AKI. Nearly 20% of AKI cases are directly or indirectly related to nephrotoxic drug exposure, including antitumor drugs (such as cisplatin) and folic acid [2, 3, 4]. Among these, Cisplatin‐induced acute kidney injury (AKI) and other chemotherapy‐related renal injuries are common complications in cancer treatment [4]. Currently, there are no effective protective measures or counterstrategies specifically for this condition. Despite significant advances in understanding the underlying mechanisms, no specific therapies for AKI have been developed [5]. Current clinical therapeutic strategies for AKI encompass supportive treatment, symptomatic treatment, and renal replacement therapy [6, 7]. However, their curative effects are largely limited. Therefore, it is crucial to identify drugs and treatment approaches which are effective at preventing kidney injury or repairing kidney injury.

Traditional Chinese herbs and their natural extracts show strong potential for disease treatment. *Salvia miltiorrhiza* is widely used in clinical practice for the treatment of various renal diseases, such as primary glomerular disease, acute and chronic renal failure, and it is an indispensable ingredient of traditional Chinese medicine compound [8]. Salvianolic acid B (SAB), one of the most abundant natural compounds found in *Salvia miltiorrhiza* , has been demonstrated to possess potent anti‐inflammatory and antioxidant effects [9, 10, 11]. Research has shown that SAB can improve kidney dysfunction by inhibiting endoplasmic reticulum stress and the PI3K/Akt/Nrf2 pathway, as well as by activating SIRT1‐mediated autophagy and Nrf2/NLRP3‐mediated pyroptosis [12]. In addition, SAB showed promising kidney‐protective effects in unilateral ureteral obstruction‐induced and ischemia/reperfusion‐induced mouse models [13, 14]. While existing studies have focused on SAB's antioxidative (e.g., Nrf2 activation) and anti‐inflammatory actions, its capacity to modulate ferroptosis‐associated pathways remains largely uncharacterized. The explicit role of SAB in cisplatin‐ or folate‐induced ferroptotic AKI models remains experimentally unvalidated, highlighting the need for further investigation given its broad spectrum of bioactivities.

Ferroptosis is an emerging form of regulated cell death characterized by iron‐dependent accumulation of lipid peroxides, leading to membrane rupture [15]. This unique modality of cell death has been implicated in various pathological processes, including neurodegenerative diseases, cancer, stroke, and AKI [16]. In the context of AKI, ferroptosis has been shown to contribute to renal tubular cell death, exacerbating tissue damage and impeding renal recovery [17, 18]. The regulation of ferroptosis involves complex interactions between glutathione peroxidase 4 (GPX4), system xc^−^ (composed of SLC7A11 and SLC3A2), and iron metabolism, with GPX4 acting as a critical antioxidant enzyme that prevents lipid peroxidation. Disruption of these pathways, such as through the inhibition of GPX4 or SLC7A11, can shift the balance toward ferroptosis.

The current study aimed to investigate the potential protective effects of SAB against AKI using both cisplatin‐induced and folic acid‐induced AKI models. Specifically, we propose that SAB enhances the activity of the SLC7A11 and GPX4 proteins by binding to peroxiredoxin 5 (PRDX5) and boosting PRDX5's bioactivity in cisplatin‐induced AKI. By targeting the activation of the PRDX5/SLC7A11‐GPX4 axis and inhibiting ferroptosis, SAB may mitigate renal tubular cell death and preserve renal function in the context of AKI. Elucidating these mechanisms could not only redefine SAB's therapeutic potential in nephrotoxic AKI but also position it as a novel scaffold for designing multifunctional ferroptosis inhibitors.

## 2 Experimental Procedures

### 2.1 Reagents

Salvianolic acid B (SAB, CAS 115939–25‐8, purity ≥ 98%), cisplatin (CAS 15663–27–1), folic acid (FAC, AS 59–30–3) and ferrostatin‐1 (fer‐1, CAS 347174–05–4) were purchased from TargetMol (Shanghai, China). Specific antibodies for KIM‐1, NGAL, GPX4, ASCL4, SLC7A11, FSP1, PRDX5, TNF‐α and IL‐β were obtained from Abclone (Wuhan, China). A specific antibody against β‐actin was purchased from ZSGB‐BIO (Beijing, China). Assay kits for malondialdehyde (MDA) and glutathione (GSH) were purchased from Beyotime Biotechnology (Jiangsu, China). Assay kits for creatinine, blood urea nitrogen (BUN), aspartate aminotransferase (AST) and alanine aminotransferase (ALT) were purchased from the Suzhou Grace Biotechnology Co. Ltd. (Jiangsu, China).

### 2.2 AKI Animal Models and Drug Administration

Eight‐week‐old male C57BL/6 mice (GemPharmatech LLC, Jiangsu, China) were used to establish AKI animal models. All mice were maintained in a pathogen‐free environment at 22°C ± 2°C and 55% ± 5% humidity in a barrier facility with 12 h light–dark cycles. All procedures involving mice and experimental protocols were approved by the Ethics Committee of Lanzhou University Second Hospital (D2024‐936).

For the cisplatin (20 mg/kg)‐induced AKI model, cisplatin was administered via intraperitoneal injection. SAB (25, 50 mg/kg; dissolved in 10% DMSO) was injected intraperitoneally 12 h before and daily after cisplatin administration, while fer‐1 (5 mg/kg) was injected intraperitoneally 30 min prior to cisplatin treatment. The normal control and model groups received the same volume of 10% DMSO as a negative control. After three days of cisplatin treatment, the animals were euthanized by the inhalation of 5% isoflurane.

For the FA (250 mg/kg)‐induced AKI model, FA dissolved in 0.3 M sodium bicarbonate was administered via intraperitoneal injection. SAB (25 mg/kg, 50 mg/kg) was injected intraperitoneally 12 h before and daily after the FA injection, while fer‐1 (5 mg/kg) was administered intraperitoneally 30 min prior to FA treatment. The normal control and model groups received the same volume of 10% DMSO as a negative control. Two days after the FA injection, the animals were euthanized by the inhalation of 5% isoflurane. Blood and kidney tissue samples were then collected for subsequent biochemical and pathological assays.

### 2.3 Cell Culture

The human renal tubular epithelial cell line HK‐2 was purchased from the Chinese Academy of Sciences Cell Bank (Shanghai, China) and maintained in our laboratory. HK‐2 cells were cultured in DMEM/F12 medium (Gibco, USA) supplemented with 10% FBS (Procell, Wuhan, China) in an incubator at 37°C with 5% CO_2_. To establish a cell damage model, HK‐2 cells were incubated with cisplatin (20 μM) for 24 h. The cells were pretreated with various concentrations of SAB (0.25, 0.5, 1, 2, 4, 8, 16, 32, or 64 μM), or with fer‐1 (0.4 μM) to assess their protective effects.

### 2.4 Cell Viability Assay

For the cell viability assay, HK‐2 cells were seeded into 96‐well plates at a density of 4 × 10^3^ cells/well and incubated at 37°C with 5% CO_2_ for 24 h. Subsequently, cells were pretreated with SAB at various concentrations for 12 h and then stimulated with cisplatin (20 μM) for an additional 24 h. Following this, 90‐μL medium and 10‐μL CCK8 solution (TargetMol, Shanghai, China) were added into each well, and the plates were incubated for 2–4 h at 37°C in the dark. Finally, the absorbance at a wavelength of 450 nm was measured using a multifunctional chemiluminescence detector (Detie, Nanjing, China).

### 2.5 RNA Extraction and Quantitative Real‐Time PCR (qRT–PCR) Analysis

Total RNA was extracted from mouse kidney tissue or HK‐2 cells using TRIzol reagent (Takara Biotechnology Co., Dalian, China). The RNA was then reverse transcribed into cDNA using the PrimeScript RT reagent kit (Servicebio, Wuhan, China) following the manufacturer's instructions. Gene expression was quantified by qRT–PCR using the SYBR Green qPCR Master Mix (Servicebio, Wuhan, China) on a real‐time thermal cycler (Bio‐Rad Laboratories Inc., USA). The PCR amplification conditions were as follows: denaturation at 95°C for 15 s, followed by annealing and elongation at 60°C for 30 s, for a total of 40 cycles. Gene expression was normalized with *β‐actin*. The sequences of the primers used are listed in Table 1.

**TABLE 1:** Primer sequences for qRT‐PCR analysis.

Gene name	Primer sequence	
	Forward (5′‐3′)	Reverse (5′‐3′)
**Gene (Human)**		
*KIM‐1*	ATCGGAAGGACACACGCTATA	ACCTTGGGTGGCACAATCT
*NGAL*	TTGGGACAGGGAAGACGA	TCACGCTGGGCAACATTA
*ACSL4*	TCTGCTTCTGCTGCCCAATT	CGCCTTCTTGCCAGTCTTTT
*COX2*	TGCTTGTCTGGAACAACTGC	TGAGCATCTACGGTTTGCTG
*GPX4*	ATCCTGGCCTTCCCGTGTAAC	CTTGCCCTTGGGTTGGATCTT
*β‐Actin*	GCACAGAGCCTCGCCTT	GTTGTCGAC GACGAGCG
**Gene (Mouse)**		
*TNF‐α*	CATCTTCTCAAAATTCGAGTGACAA	TGGGAGTAGACAAGGTACAACCC
*IL‐1β*	CTTTGAAGTTGACGGACCC	TGAGTGATACTGCCTGCCTG
*MCP‐1*	CTTCTGGGCCTGCTGTTCA	CCAGCCTACTCATTGGGATCA
*GPX4*	CATGCCCGATATGCTGAGTGTGG	TGCTAGGAGCCAGAGCAGTA
*ACSL4*	ACTTACCTTTGGCTCATG	CAGTACAGTACAATCACCCT
*SLC7A11*	TACTGACAAACGTGGCCTATT	AACCTGGAGACAGCGAACACA
*COX2*	CACACTCTATCACTGGCACC	TCCAGGAGGATGGAGTTGTT
*β‐Actin*	AGTGTGACGTTGACATCCGT	TGCTAGGAGCCAGAGCAGTA

### 2.6 Immunofluorescence (IF) Assay

Adherent cells were fixed with 4% paraformaldehyde and blocked with 2% BSA. They were then incubated with KIM‐1 antibody overnight at 4°C, followed by incubation with the corresponding fluorescent secondary antibody at 37°C for 1 h. Subsequently, the nuclei were stained with DAPI. The fluorescence signals were detected and visualized using a fluorescence microscopy (Nexcope, Jiangsu, China).

### 2.7 Protein Extraction and Western Blot

The western blot assay was performed as previously described in our articles [19, 20]. Total protein was extracted from mouse kidney tissue or human HK‐2 cells using ice‐cold RIPA lysis buffer (Solarbio, Shanghai, China) supplemented with 1% PMSF (Solarbio, Shanghai, China). Following centrifugation and denaturation by boiling, equal amounts of protein (50 –100 μg) were subjected to 10%–12% SDS‐PAGE and electroblotted onto PVDF membranes (0.45 μM, Sigma‐Aldrich Chemicals, USA). The membranes were blocked with QuickBlock Blocking Buffer (Beyotime Biotechnology, Jiangsu, China) for 1 h at room temperature. Subsequently, the membranes were incubated with the appropriate primary antibody for 3 h at room temperature. The membranes were cut prior to hybridization with antibodies during the western blot procedure. Then, the membranes were washed three times with 1 × TBST and incubated with dye‐labeled secondary antibodies (1:10000) (Odyssey IRDye 800 or IRDye 700, LI‐COR, USA) for 1.5 h at room temperature. Finally, the membranes were washed again and visualized using a two‐color infrared fluorescence imaging system (Odyssey Clx, LI‐COR, USA) and quantified using the Image Studio Ver 3.1 software (Li‐Cor, USA).

### 2.8 Lipid Peroxidation Assay

Lipid peroxidation levels were assessed using BODIPY 581/591 C11 dye (Beyotime Biotechnology, Jiangsu, China). The reagent was applied to the cell membranes of living cells. Following peroxide oxidation, the peak wavelength of the fluorescence emission shifted from 590 to 510 nm, resulting in a change in fluorescence color from red to green in the living cells. The fluorescence signals were detected using fluorescence microscopy (Nexcope, Jiangsu, China).

### 2.9 MDA and GSH Levels Detection

The expression levels of MDA and GSH in HK‐2 cells and kidney tissues were measured using MDA and GSH activity assay kits, respectively, according to the manufacturer's protocol.

### 2.10 Masson and Hematoxylin–Eosin (HE) Staining

Pathological staining in the collected kidney tissues was performed using Masson staining kits (Solarbio, China), and histological changes in the collected kidney tissues were evaluated using the HE staining kits (Beyotime Biotechnology, Jiangsu, China). Two researchers independently observed and evaluated histopathological changes back‐to‐back.

### 2.11 Immunohistochemical (IHC) Assay

For immunohistochemical staining, the kidney tissues were embedded in paraffin and sectioned into 4‐μm‐thick slices. After blocking with 1% BSA, the slices were incubated with primary antibodies overnight at 4°C. Following three washes with TBST, the slices were incubated with a horseradish peroxidase–polymer labeled secondary antibody for 30 min and then subjected to DAB staining. The sections were subsequently observed under a microscope (OLYMPUS, Japan).

### 2.12 Target Prediction

We used the PharMapper databases online database (http://lilab‐ecust.cn/pharmmapper/index.html) [21], with the species limited to “ *Homo sapiens* ” to predict the targets of SAB.

### 2.13 Cellular Thermal Shift Assay (CETSA)

HK‐2 cells were incubated with either DMSO or 10 μM SAB for 1 h and then washed three times with PBS. The cells were suspended in 1 mL of PBS and divided into equal volumes, followed by heating for 10 min at the specified temperatures. After cooling to room temperature for 10 min, SDS‐PAGE loading buffer was added, and the samples were immediately boiled for western blot analysis.

### 2.14 Drug Affinity Responsive Target Stability (DARTS)

In brief, approximately 1 × 10^7^ HK‐2 cells were lysed on ice for 30 min using NP‐40 extraction reagent (Beyotime Biotechnology, Jiangsu, China). The cell lysates were then quantitated using a bicinchoninic acid (BCA) assay and incubated with SAB (10 μM) for 1.5 h at room temperature. Following this, the lysates were digested with pronase at ratios of 1:600, 1:400, 1:200, and 1:150 (w/w) for 30 min. Loading buffer was then added to the lysates, and the samples were boiled for 5 min. The resulting samples were separated by SDS‐PAGE and incubated with antibodies specific to the target protein.

### 2.15 Molecular Docking

AutoDockTools (version 1.5.6) was utilized for hydrogenation and charge calculations of the core protein. The active structures of smallmolecules were downloaded from the PubChem database and processed using AutoDockTools. Finally, receptor and ligand docking was performed using AutoDock Vina (version 1.1.2).

### 2.16 Silencing of PRDX5 in HK‐2 Cells

To silence *PRDX5* expression, HK‐2 cells were transiently transfected with *PRDX5*‐specific siRNA and negative control siRNA (NC) which were designed and synthesized by Tsingke. The sequences of *PRDX5* siRNAs were listed in Table 2. Lipofectamine 2000 reagent (Invitrogen, 11 668‐019, USA) was used for transfection according to the manufacturer's instructions. The diluted siRNA was mixed with Lipofectamine 2000 and incubated at 37°C for 20 min before being added to the HK‐2 cells. After transfection for 6 h, the cells were cultured in fresh medium containing 10% FBS for an additional 18 h. Subsequently, cells were treated with SAB (2 μM) or cisplatin (20 μM). Cells were then harvested for western blot, as well as for the detection of MDA and GSH levels.

**TABLE 2:** The sequences of *PRDX5* siRNA and negative control siRNA.

siRNA	Sequences
siRNA1	sense: 5’‐GGUUCUCCAUGGUGGUACA‐3 antisense: 5′‐UGUACCACCAUGGAGAACC‐3′
siRNA2	sense: 5′‐GGAAGGAGACAGACUUAUU‐3′ antisense: 5′‐AAUAAGUCUGUCUCCUUCC‐3′
siRNA3	sense: 5′‐CAGCAAGACGGUACAGUGA‐3′ antisense: 5′‐UCACUGUACCGUCUUGCUG‐3′
Negative control siRNA	sense: 5′‐UUCUCCGAACGUGUCACGUTT‐3′ antisense: 5′‐ACGUGACACGUUCGGAGAATT‐3′

### 2.17 Statistical Analysis

The data were presented as mean ± SEM in this study. The statistical significance levels of the differences between two groups were tested by two‐tailed Student's t‐tests. For multiple groups, one‐way ANOVA followed by Tukey's multiple comparisons test was utilized to detect statistical significance. Values of *p* < 0.05 were considered significant. The statistical analyses were performed using GraphPad Prism 9.0.

## 3 Results

### 3.1 SAB Treatment Mitigates Cisplatin‐Induced Damage in HK‐2 Cell

**FIGURE 1:**
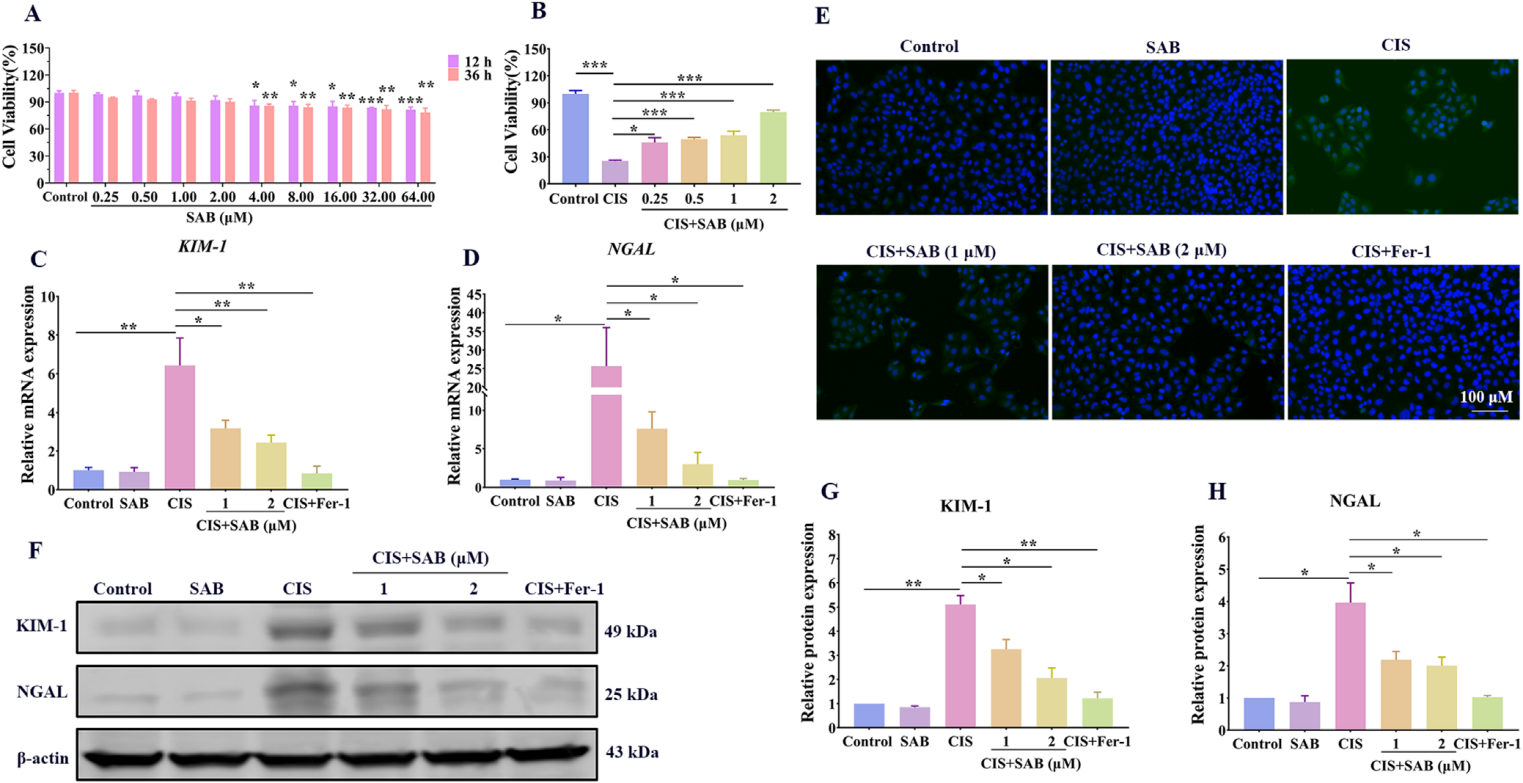
SAB treatment mitigates cisplatin‐induced damage in HK‐2 cells. (A) Cell viability assay of SAB in HK‐2 cells. (B) SAB restored cell viability after stimulation with cisplatin. (C‐D) qRT–PCR analysis of *KIM‐1 and NGAL*. (E) Immunofluorescence analysis of KIM‐1. (F‐H) Western blot analysis of KIM‐1 and NGAL. The results are presented as mean ± SEM, and all experiments were repeated three times. *p < 0.05; **p < 0.01; ***p < 0.001. SAB, Salvianolic acid; Cis, Cisplatin.

The cytotoxic effect of SAB was assessed using the CCK8 assay, and the results indicated that when the concentration of SAB exceeded 2 μM, the growth status of HK‐2 cells was affected (Figure 1A). To investigate whether SAB has a protective effect, the HK‐2 cells were pretreated with various concentrations of SAB and then incubated with 20 μM cisplatin. The CCK8 results demonstrated that SAB inhibited cisplatin‐induced cell death in HK‐2 cells in a dose‐dependent manner (Figure 1B). We then measured the mRNA and protein expression levels of KIM‐1 and NGAL (two markers of renal tubular injury). The qRT‐PCR results showed that increasing trends of KIM‐1 and NGAL induced by cisplatin were significantly abolished by SAB pretreatment (Figure 1C,D). The IF result of KIM‐1 and western blot results were consistent with the qRT‐PCR results (Figure 1E–H).

### 3.2 SAB Treatment Alleviates Cisplatin‐Induced Ferroptosis and Lipid Peroxidation in HK‐2 Cells

**FIGURE 2:**
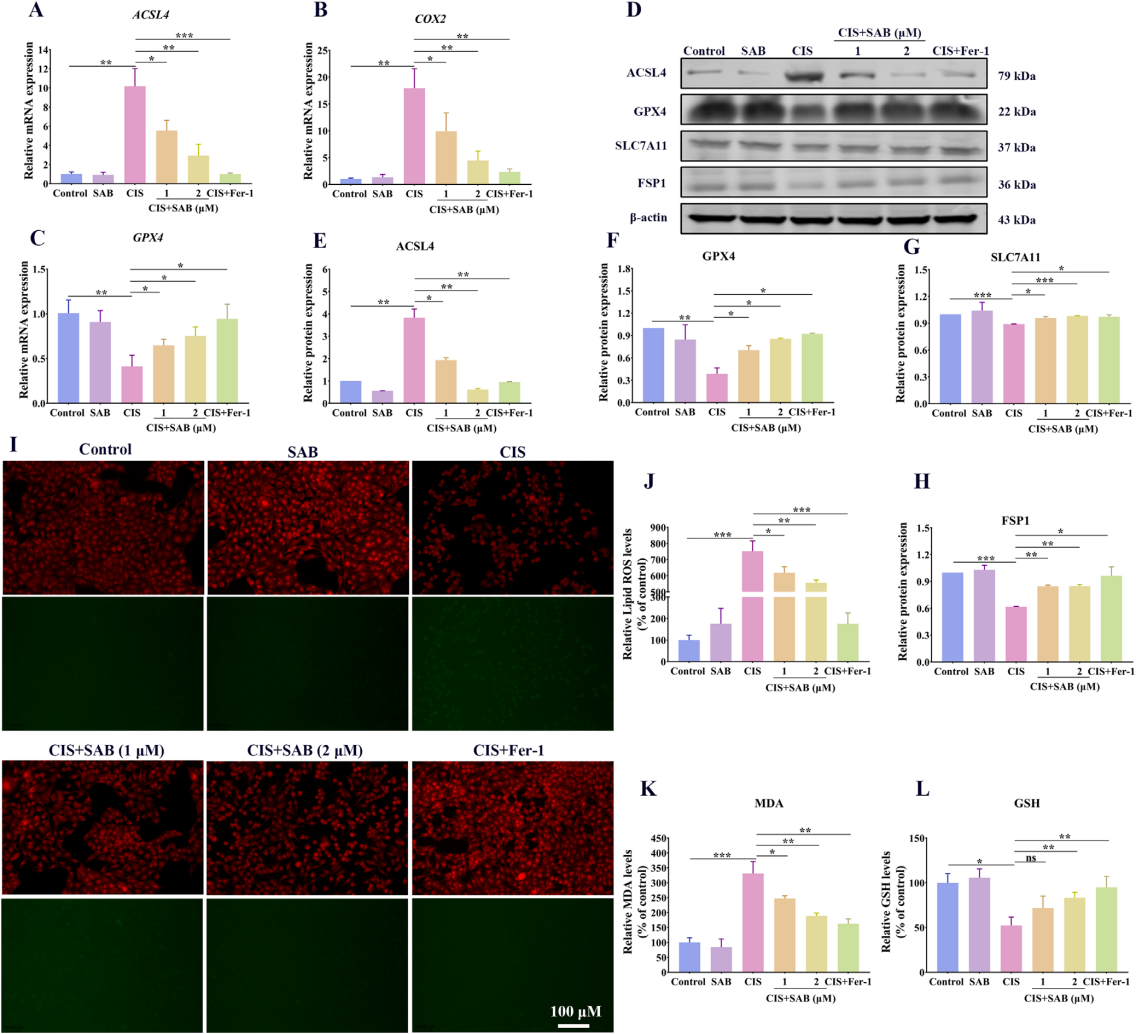
SAB treatment alleviates cisplatin‐induced ferroptosis and lipid peroxidation in HK‐2 cells. (A‐C) qRT‐PCR analysis of *ACSL4, COX2*, *and GPX4*. (D‐H) Western blot analysis of ACSL4, GPX4, SLC7A11, and FSP1. (I‐J) Lipid ROS staining and corresponding statistical analysis of fluorescence intensity (scale bar = 100 μm). (K) Detection of MDA levels. (L) Detection of GSH levels. The results are presented as mean ± SEM, and all experiments were repeated three times. *p < 0.05; **p < 0.01; ***p < 0.001, ns, not significant.

Ferroptosis is a form of cell death driven by iron‐dependent lipid peroxidation. The glutathione peroxidase 4 (GPX4) pathway is a well‐known regulator of ferroptosis [22]. ACSL4 and COX‐2 promote the synthesis of lipid peroxides, while GPX4 facilitates the degradation of these lipid peroxides [2]. The qRT‐PCR or western blot results showed that both SAB and Fer‐1 reduced the cisplatin‐induced upregulation of ACSL4 and COX‐2 (Figure 2A,B,D,E), while restoring the cisplatin‐induced downregulation of GPX4, SLC7A11, and FSP1 (Figure 2C,D,F,H). To assess lipid peroxidation, we analyzed lipid ROS in cisplatin‐treated HK‐2 cells. Fluorescence microscopy revealed that SAB significantly reduced the lipid ROS accumulation caused by cisplatin (Figure 2I,J). Furthermore, our study showed that SAB restored GSH levels and alleviated MDA accumulation (Figure 2K,L). Taken together, the above results suggest that SAB effectively inhibits cisplatin‐induced ferroptosis and lipid peroxidation in renal tubular cells.

### 3.3 SAB Treatment Alleviates Cisplatin‐Induced AKI in Mice

**FIGURE 3:**
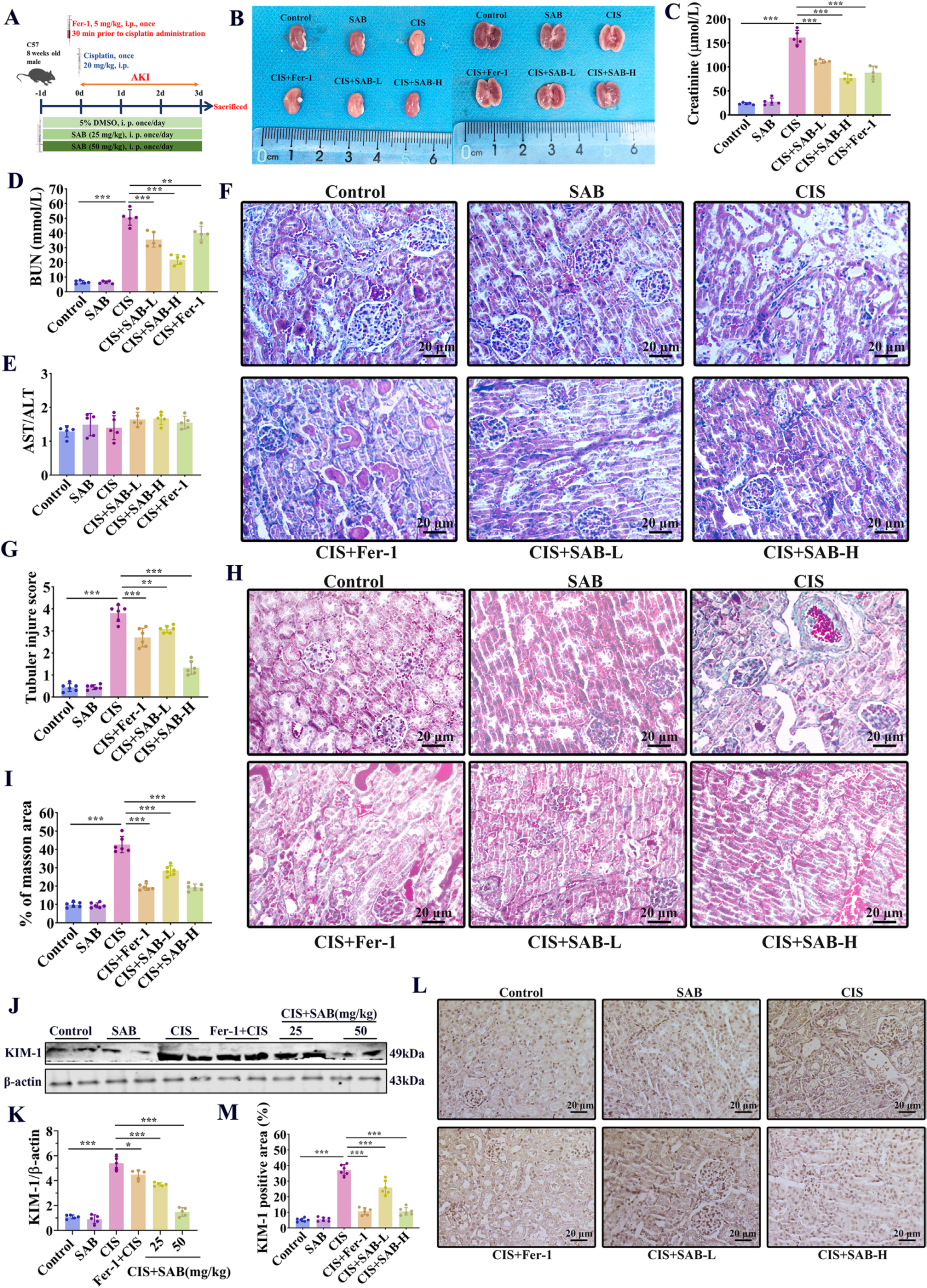
SAB protects against renal dysfunction and pathological damage in cisplatin‐induced AKI mice. (A) The schematic diagram of experimental design. (B) Representative gross‐morphological images of kidney cross section. (C‐E) The creatinine, BUN, and ALT/AST levels (*n* = 6). (F‐G) HE staining of kidney sections and tubular injury score. (H‐I) Masson staining of kidney sections and corresponding statistical analysis. (J‐K) WB analysis of KIM‐1 and corresponding statistical analysis. (L‐M) Immunohistochemical analysis of KIM‐1 and corresponding statistical analysis. *p < 0.05; **p < 0.01; ***p < 0.001.

To further verify whether SAB exerts protective effects in vivo, we performed relevant experiments in a cisplatin‐induced AKI mouse model (Figure 3A,B). Kidneys in the cisplatin group pronounced white discoloration and edema, whereas SAB or Fer‐1 administration improved their appearance (Figure 3B). By testing serum samples, we found that SAB alleviated the increases in serum creatinine and BUN levels induced by cisplatin in a dose‐dependent manner (Figure 3C,D). Additionally, we quantified the levels of ALT and AST in both the control and SAB groups. There was no statistically significant difference in AST/ALT levels between the two groups, indicating that SAB does not exert a toxic effect on the mouse liver in vivo (Figure 3E). Subsequently, we performed HE staining to observe the effect of SAB on cisplatin‐induced kidney damage. HE staining revealed dilated tubules and lysis of tubular cells in the AKI model group, while SAB alleviated these pathological changes (Figure 3F,G). Masson staining further confirmed that SAB mitigated cisplatin‐induced renal pathological damage (Figure 3H,I). Moreover, western blot and immunohistochemistry (IHC) assays showed that the expression level of KIM‐1 in kidney tissue increased in the cisplatin group, indicating increased renal injury. In contrast, the SAB group exhibited a reduction in renal injury (Figure 3J,M). Collectively, these results indicated that SAB had a therapeutic effect comparable to that of the ferroptosis inhibitor Fer‐1.

### 3.4 SAB Treatment Alleviates Cisplatin‐Induced Renal Inflammatory Response in Mice

**FIGURE 4:**
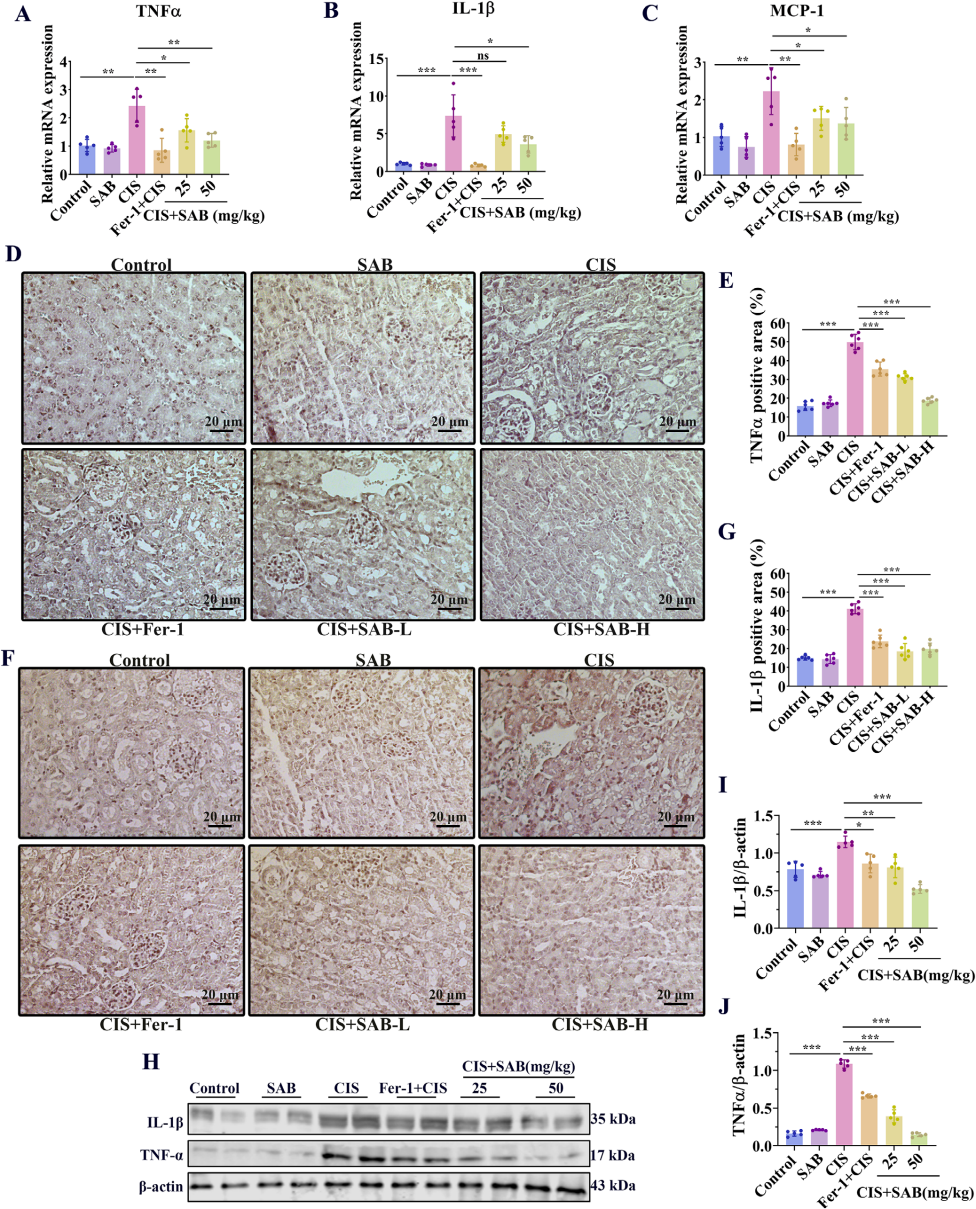
SAB treatment alleviates cisplatin‐induced renal inflammatory response in mice. (A‐C) qRT‐PCR analysis of *TNF‐α*, *IL‐1β* and *MCP‐1*. (D‐E) Immunohistochemical analysis of TNF‐α and corresponding statistical analysis. (F‐G) Immunohistochemical analysis of IL‐1β and corresponding statistical analysis. (H‐J) WB analysis of TNF‐α and IL‐1β and corresponding statistical analysis. *p < 0.05; **p < 0.01; ***p < 0.001, ns, not significant. *n* = 6.

Since inflammation is a common pathological feature of AKI, we next examined the effect of SAB on the inflammatory response of the kidneys in cisplatin‐induced AKI mice. The qRT–PCR results showed that cisplatin stimulation significantly increased the mRNA levels of inflammation‐related cytokines, such as *TNF‐α*, *IL‐1β*, and *monocyte chemoattractant protein‐1 (MCP‐1)*. However, SAB and fer‐1 pretreatment significantly reduced the mRNA levels of these cytokines (Figure 4A,C). Consistently, the immunohistochemical analysis revealed that cisplatin significantly increased the levels of TNF‐α and IL‐1β, while SAB and fer‐1 treatment dose‐dependently decreased the levels of these inflammatory factors (Figure 4D–G). Furthermore, the upregulated protein levels of TNF‐α and IL‐1β induced by cisplatin were markedly rescued by SAB and fer‐1 treatment (Figure 4H–J). These findings indicated that SAB treatment ameliorated the renal inflammatory response in cisplatin‐induced AKI.

### 3.5 SAB Treatment Inhibits Ferroptosis in Cisplatin‐Induced AKI Mice

**FIGURE 5:**
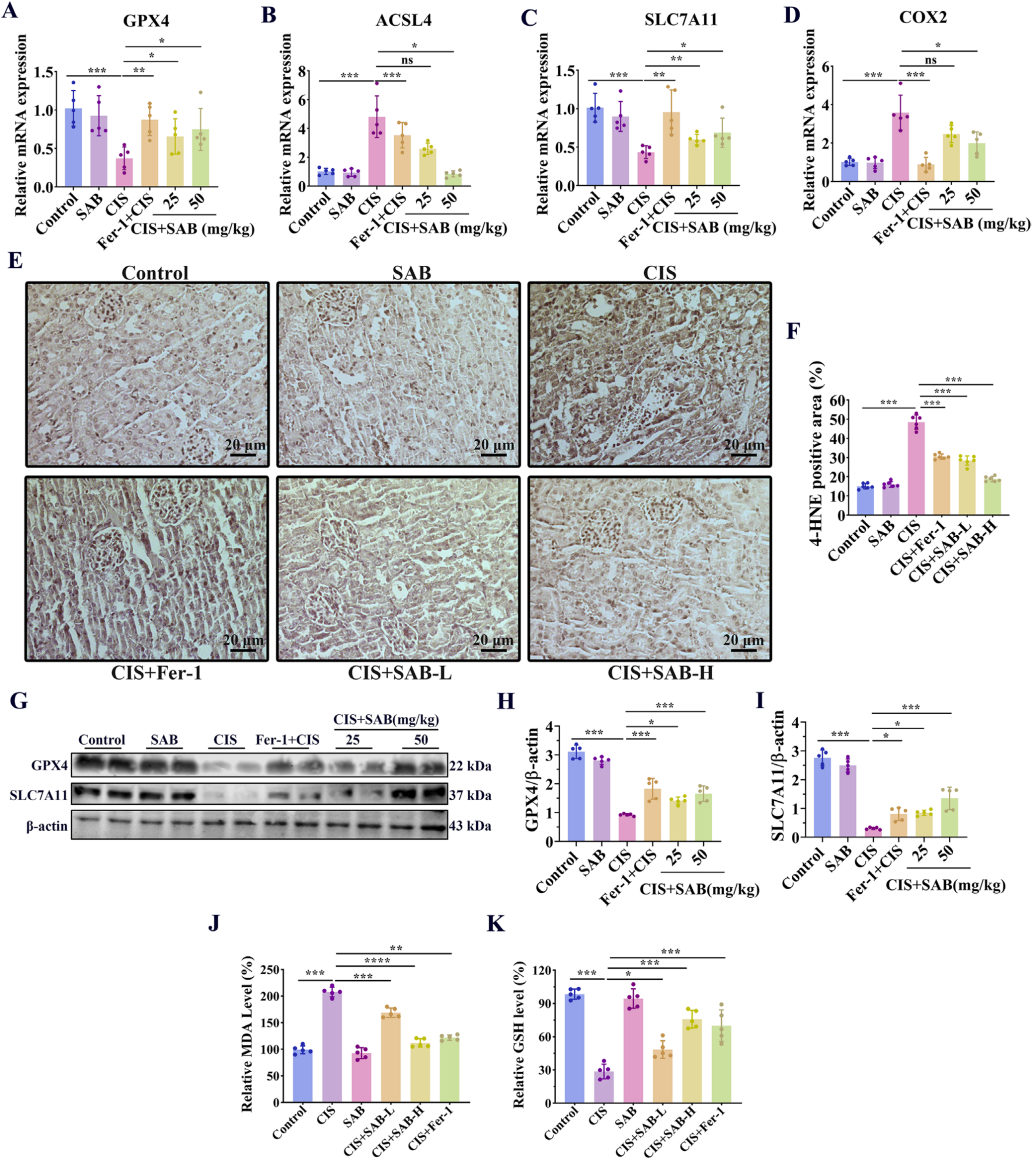
SAB treatment inhibits ferroptosis in cisplatin‐induced AKI mice. (A‐D) qRT‐PCR analysis of *GPX4*, *ACSL4, SLC7A11*, and *COX‐2*. (E‐F) Immunohistochemical analysis of 4‐HNE and corresponding statistical analysis. (G‐I) WB analysis of GPX4 and SLC7A11 and corresponding statistical analysis. (J) Detection of MDA levels. (K) Detection of GSH levels. *p < 0.05; **p < 0.01; ***p < 0.001, ns, not significant. *n* = 6.

We next investigated whether the protective effect of SAB in cisplatin‐induced AKI was related to ferroptosis. We found that SAB and fer‐1 alleviated the cisplatin‐induced down‐regulation of *GPX4* and *SLC7A11* and up‐regulation of *ACSL4* and *COX‐2* (Figure 5A–D). Immunohistochemical analysis showed that SAB and fer‐1 reduced the accumulation of 4‐HNE in the cisplatin‐induced animal model (Figure 5E,F). The protein levels of GPX4 and SLC7A11 were consistent with their mRNA levels in the cisplatin‐induced AKI model (Figure 5G–I). Additionally, SAB and fer‐1 alleviated the cisplatin‐induced accumulation of MDA levels (Figure 5J) and the depletion of GSH levels (Figure 5K). These findings implied that the inhibitory effect of SAB on ferroptosis could be a key mechanism through which it improves cisplatin‐induced AKI.

### 3.6 PRDX5 May Be the Main Target Through Which SAB Regulates Ferroptosis

**FIGURE 6:**
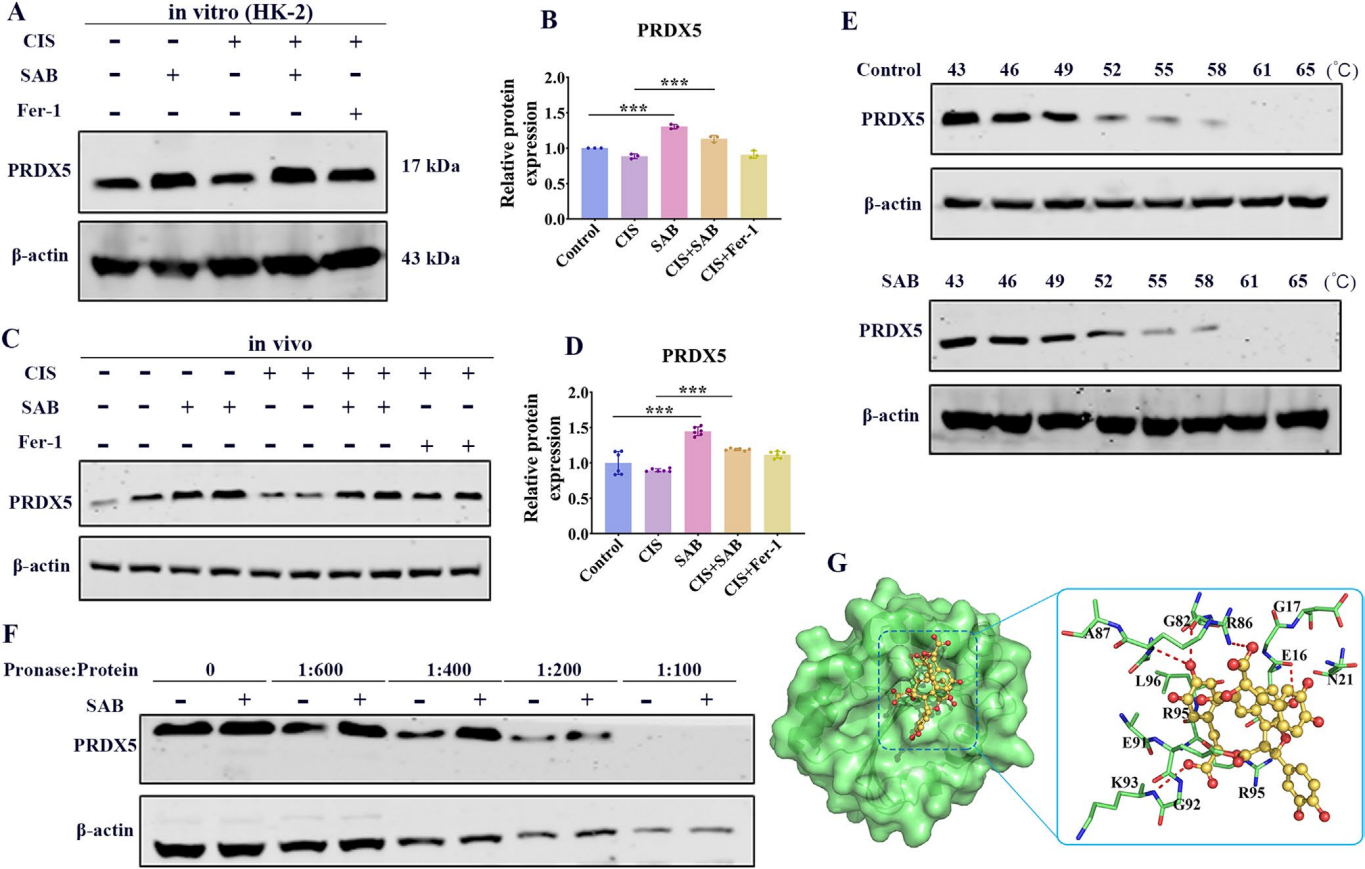
PRDX5 plays a critical role in SAB‐mediated inhibition of ferroptosis. (A‐B) Western blot analysis of PRDX5 in HK‐2 cells and corresponding statistical analysis. (C‐D) Western blot analysis of PRDX5 in AKI mice and corresponding statistical analysis. (E) CETSA‐Western blot analysis showed the protection of PRDX5 by SAB at different temperature gradients. (F) DARTS‐Western blot analysis showed the resistance of PRDX5 to pronase digestion under the treatment of SAB. (G) Results of molecular docking. The results are presented as mean ± SEM, and all experiments were repeated three times. ***p < 0.001.

To predict the target proteins of SAB, we used the online database, PharMapper, and finally identified potential therapeutic targets of SAB. Notably, PRDX5 has been reported to regulate ferroptosis [23]. We assessed the expression levels of PRDX5 in vivo and in vitro and found that SAB significantly increased PRDX5 expression in the context of cisplatin treatment (Figure 6A–D). Therefore, we concluded that SAB mainly exerted its inhibitory effect on ferroptosis through PRDX5. CETSA and DARTS methods were used to detect the binding efficiency of SAB with PRDX5. The CETSA results indicated that PRDX5 exhibited significant thermal stabilization upon SAB treatment (Figure 6E). Consistently, the DARTS results also demonstrated that SAB significantly enhanced the stability of PRDX5 during pronase‐induced degradation (Figure 6F). Subsequently, we conducted molecular docking analysis between SAB and PRDX5 and found that SAB formed hydrogen bonds with G82, R86, K93, and E16 of PRDX5 (Figure 6G). The binding energy values predicted by molecular docking were—7.60 kcal/mol, suggesting a strong interaction between SAB and PRDX5. These findings indicate that PRDX5 is a potential target of SAB.

### 3.7 SAB Inhibits Cisplatin‐Induced Ferroptosis in HK‐2 Cells Through an PRDX5‐Dependent Mechanism

**FIGURE 7:**
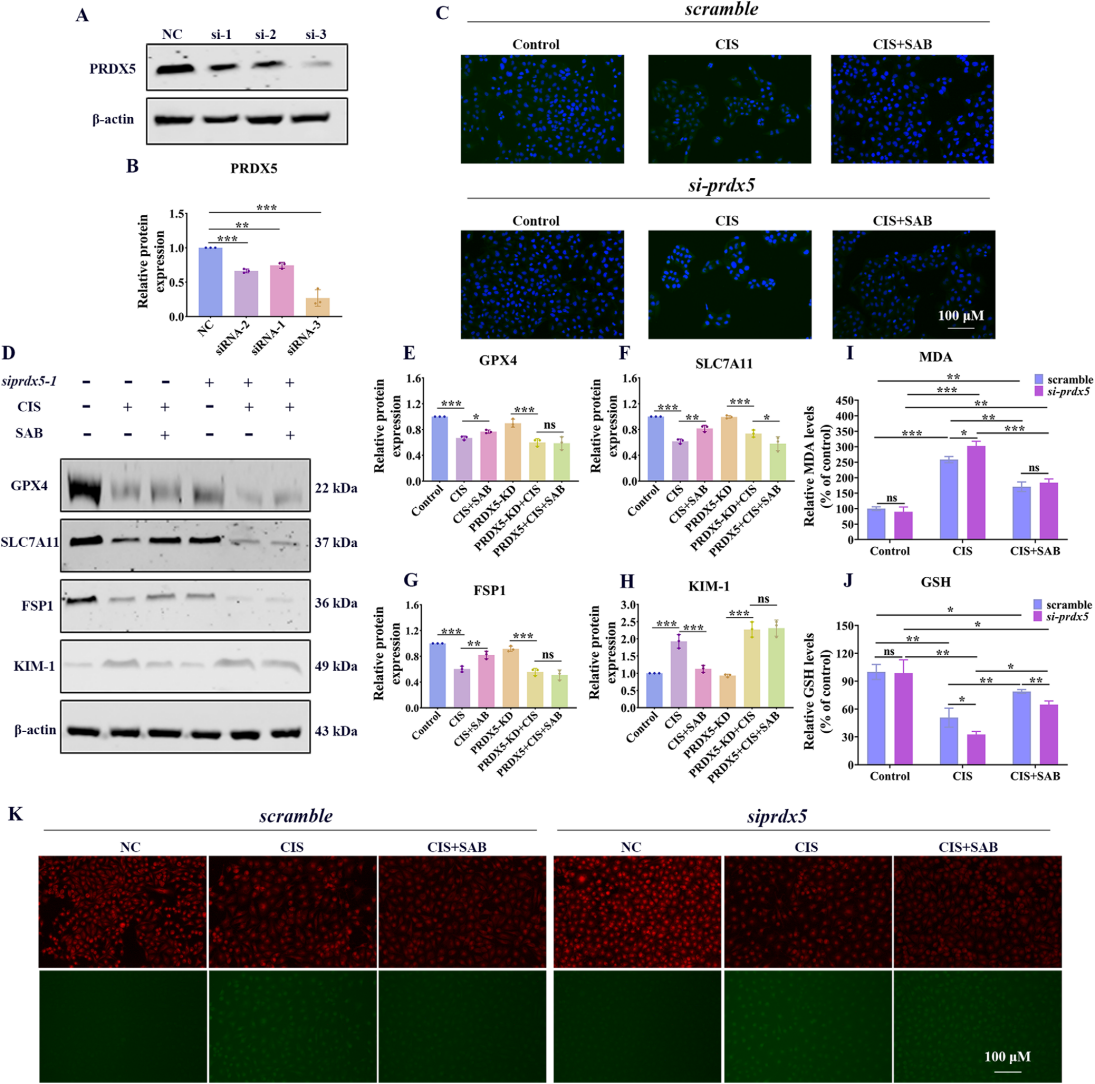
SAB inhibits cisplatin‐induced ferroptosis in HK‐2 cells through a PRDX5‐dependent mechanism. (A‐B) Validation of the efficiency of PRDX5 silencing. (C) Immunofluorescence analysis of KIM‐1. (D‐H) Western blot analysis of GPX4, SLC7A11, FSP1, and KIM‐1. (I) Detection of the MDA content. (J) Detection of the GSH content. (K) Detection of the lipid ROS levels. The results are presented as mean ± SEM, and all experiments were repeated three times. *p < 0.05; **p < 0.01; ***p < 0.001, ns, not significant.

To further validate that the protective effect of SAB is dependent on PRDX5, we silenced *PRDX5* in HK‐2 cells. The knockdown efficiency of *PRDX5* by siRNA was confirmed by western blot. As expected, *PRDX5* siRNA significantly diminished the protein expression of PRDX5 (Figure 7A,B). The results of IF and western blot revealed that silencing PRDX5 significantly reduced the protein expression of cisplatin‐induced KIM‐1, but SAB did not further downregulate KIM‐1 expression (Figure 7C,D,H). Additionally, SAB‐induced upregulation of GPX4, SLC7A11, and FSP1 in cisplatin‐treated HK‐2 cells was abolished after PRDX5 knockdown (Figure 7D–G). Furthermore, PRDX5 knockdown partially reversed the increase in GSH and the decrease in MDA levels induced by SAB treatment (Figure 7I,J). Moreover, the reduction in lipid ROS levels caused by SAB in cisplatin‐treated cells was also restored by PRDX5 knockdown (Figure 7K). These findings indicate that PRDX5 is a critical target for the ferroptosis‐inhibitory effect of SAB in the kidney.

### 3.8 SAB Treatment Alleviates Injury of Renal Tubular Epithelial Cells in FA‐Induced AKI Mice

We also established an AKI mouse model through intraperitoneal injection of FA to investigate the protective effect of SAB (Figure S1B). Consistent with the findings from the cisplatin‐induced AKI mouse model, SAB and fer‐1 alleviated the increases in serum creatinine and BUN levels induced by FA (Figure S1D). There was no statistical significance in AST/ALT levels between the two groups (Figure S1E). HE and Masson staining showed that SAB and fer‐1 treatment mitigated FA‐induced renal pathological damage. Additionally, Masson staining further confirmed that SAB alleviated cisplatin‐induced renal pathological damage (Figure S1I). Furthermore, western blot and IHC assay revealed that the expression level of KIM‐1 in the kidney tissue was elevated in the FA group, indicating increased renal injury by FA. In contrast, the SAB group exhibited a reduction in renal injury (Figure S1M). Collectively, these results indicated that SAB plays a protective role in FA‐induced AKI.

### 3.9 SAB Treatment Alleviates FA‐Induced Renal Inflammatory Response in Mice

Consistent with the findings in the cisplatin‐induced AKI mouse model, the qRT–PCR results showed that FA stimulation significantly increased the mRNA levels of inflammation‐related cytokines, *TNF‐α*, *IL‐1β*, and *MCP‐1*. However, pretreatment with SAB and fer‐1 significantly reduced the mRNA levels of these cytokines (Figure S2A–C). The immunohistochemical analysis revealed that FA significantly increased the levels of TNF‐α and IL‐1β, while SAB treatment decreased the levels of these inflammatory factors (Figure S2D–G). Additionally, the upregulated protein levels of TNF‐α and IL‐1β induced by FA were markedly rescued following treatment with SAB and fer‐1 (Figure S2H–J). These results indicated that SAB treatment ameliorated the renal inflammatory response in FA‐induced AKI.

### 3.10 SAB Inhibits FA‐Induced Lipid Peroxidation and Ferroptosis in AKI Mice

We also investigated whether the protective effect of SAB in FA‐induced AKI was associated with ferroptosis. Our results showed that SAB alleviated the FA‐induced down‐regulation of *GPX4* and *SLC7A11* and up‐regulation of *ACSL4* and *COX‐2* (Figure S3A–D). Immunohistochemical analysis revealed that SAB reduced the accumulation of 4‐HNE in the FA‐induced animal model (Figure S3F). Furthermore, SAB alleviated the FA‐induced down‐regulation of GPX4, SLC7A11, FSP1, and PRDX5 (Figure S3G–K). Moreover, SAB reduced the FA‐induced accumulation of MDA levels (Figure S3L) and the depletion of GSH levels (Figure S3M). These results collectively suggested that SAB can alleviate FA‐induced lipid peroxidation and ferroptosis in renal tubular epithelial cells.

## 4 Discussion

Salvianolic acid B, derived from *Salvia miltiorrhiza* Bunge, exhibits antioxidant, anti‐inflammatory, and antifibrotic bioactivities across various tissue injury disease models, including pulmonary fibrosis, hepatic fibrosis, myocardial ischemia/reperfusion injury, and cerebral ischemia–reperfusion injury, etc. [24, 25, 26, 27]. Recently, studies have shown that SAB can inhibit cell death by suppressing ferroptosis and downregulating apoptosis [10, 28]. Cell death, inflammation, and fibrosis exacerbate the pathological condition of AKI [29, 30]. However, the role of SAB in AKI remains unknown. In this study, we aim to demonstrate the therapeutic effects and mechanisms of SAB in the context of cisplatin and FA‐induced AKI. Our findings indicate that SAB can mitigate cisplatin‐induced damage in HK‐2 cells by inhibiting ferroptosis. The in vivo study also demonstrated its protective effects against AKI induced by cisplatin and FA. Mechanistically, we discovered that SAB binds to PRDX5 and enhances its antioxidant activity.

Ferroptosis, distinct from other forms of programmed cell death (PCD) at both the morphological and biochemical levels, is characterized by iron‐ and reactive oxygen species (ROS)‐dependent lipid peroxidation, ultimately leading to membrane rupture and cell death [31, 32]. Strong evidence suggests that ferroptosis plays a crucial role in the development of various organ injuries and degenerative diseases [33, 34]. For instance, ischemia‐induced activation of ACSL4 contributes to ferroptosis, which plays a crucial role in tissue injury during intestinal ischemia/reperfusion. Inhibiting ACSL4‐mediated ferroptosis alleviates tissue injury caused by ischemia/reperfusion in the intestine [35]. Conditional ablation of glutathione peroxidase 4 (GPX4), an antioxidant enzyme essential for preventing ferroptosis by repairing oxidative damage to lipids in the neurons of adult mice, leads to the rapid onset and progression of paralysis and eventual death [36]. Ferroptosis also contributes to drug‐induced tissue injury. For example, the inducers of pulmonary fibrosis and injury, such as bleomycin (BLM) and lipopolysaccharide (LPS), trigger ferroptosis in lung epithelial cells [37]. Inhibiting ferroptosis with the iron chelator deferoxamine (DFO) or the ferroptosis inhibitor liproxstatin‐1 (Lip‐1) can alleviate lung injury induced by bleomycin or LPS. Ferroptosis also contributes to the pathology of AKI. Liang's research demonstrated that cisplatin treatment induces renal cell ferroptosis by exacerbating mitochondrial DHODH acetylation, depleting CoQH2, and promoting lipid peroxidation [38]. Additionally, cisplatin elevates renal iron levels and facilitates iron‐catalyzed ferroptosis in renal cells [39, 40].

**FIGURE 8:**
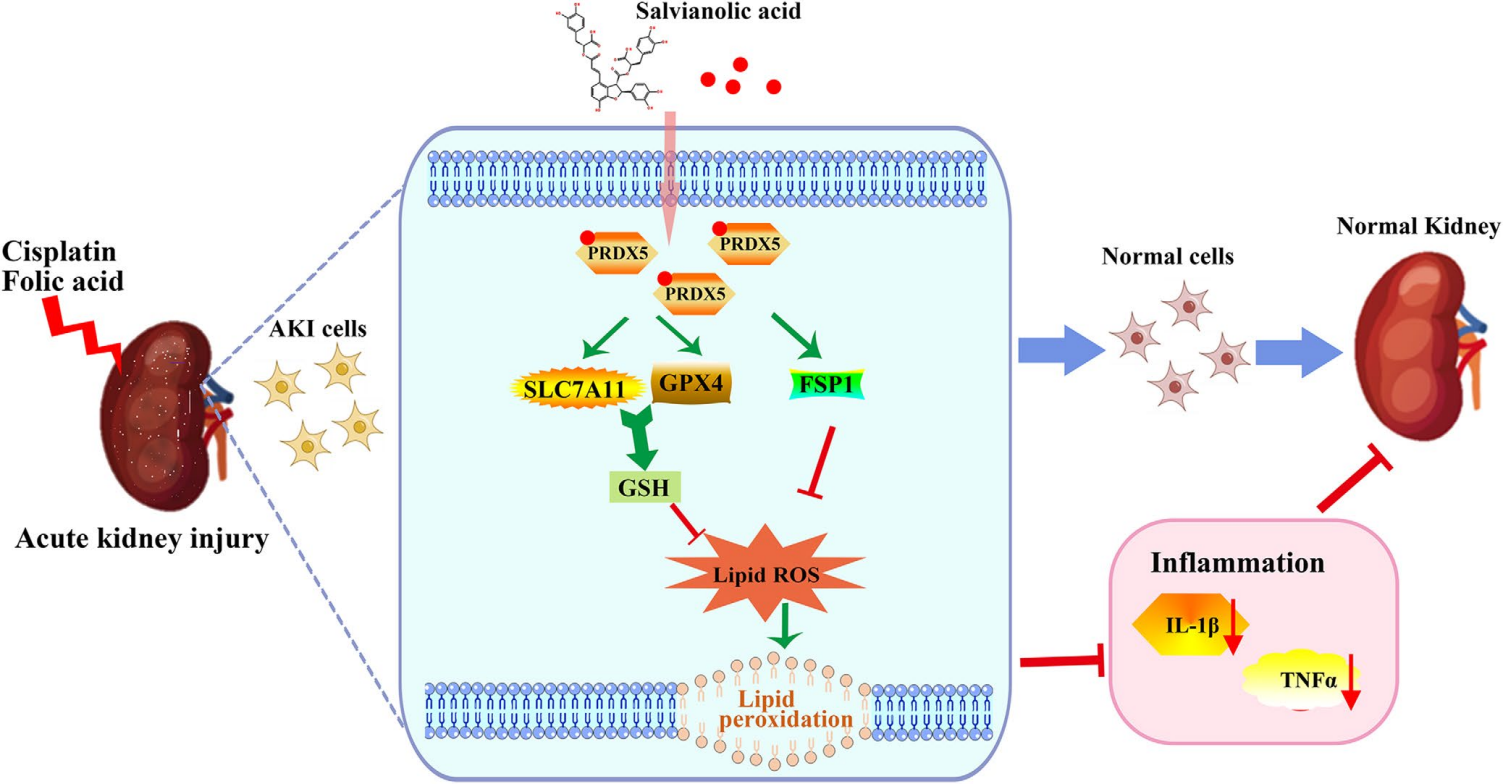
Mechanistic diagram of SAB‐mediated alleviation of AKI through the regulation of PRDX5‐mediated ferroptosis in renal tubular epithelial cells.

Peroxiredoxins (PRDXs) are intracellular enzymes that scavenge hydrogen peroxide (H_2_O_2_) by donating an electron from the peroxidatic cysteine located at their amino terminus [41]. Recent studies have found that PRDXs are involved in regulating the occurrence of ferroptosis in cells. During ferroptotic stress, PRDX3 undergoes hyperoxidation and translocates from the mitochondria to the plasma membrane, where its presence inhibits cystine uptake, thereby promoting ferroptotic damage [42, 43]. PRDX6 was found to inhibit ferroptosis by enhancing selenium utilization [44, 45, 46, 47]. PRDX1 participates in the inhibition of ferroptosis by enhancing the stability of the NRF2 protein and promoting NRF2‐mediated transcription of GPX4 [48]. PRDX5 could protect ethyl β‐carboline‐3‐carboxylate‐induced cell death. Furthermore, studies also suggested that PRDXs correlated with AKI. Overexpression of PRDX6 inhibits renal apoptosis and leukocyte infiltration, leading to a reduction in LPS‐induced AKI by decreasing LPS‐induced ROS concentrations in the kidney [49]. However, it remains unclear whether PRDXs can regulate AKI by inhibiting ferroptosis. In our study, we discovered that SAB significantly upregulated PRDX5 expression in cisplatin and folic acid‐treated renal tubular epithelial cells, coinciding with attenuated oxidative damage markers (e.g., MDA), demonstrating that PRDX5 is involved in the regulation of ferroptosis and inflammation. Knockdown of PRDX5 can abolish SAB's protective effects proven by exacerbating MDA generation while inhibiting the production of GSH in HK‐2 cells, confirming PRDX5's indispensability in this context. Based on the findings mentioned above, PRDX5 could be a promising therapeutic target for the treatment of AKI. (Figure 8).

Our study is the first to reveal the therapeutic potential of SAB in AKI. Additionally, we have identified PRDX5 as a target of SAB and demonstrated that PRDX5 can inhibit cisplatin‐ and FA‐induced AKI. These findings enhance our understanding of the mechanisms underlying AKI and suggest that SAB may serve as a promising therapeutic agent for preventing or treating AKI in patients undergoing cisplatin therapy. Future studies are needed to further investigate the therapeutic potential of SAB in AKI and to explore additional mechanisms by which it exerts its protective effects.

## Author Contributions

Yan Tao: conceptualization; methodology; formal analysis; data curation; funding acquisition; writing – original draft. Shengjun Fu: conceptualization; methodology; formal analysis; data curation. Jianzhong Lu: methodology; formal analysis; data curation; funding acquisition. Beitang Fu: methodology; data curation; formal analysis. Shanhui Liu: conceptualization; methodology; formal analysis; data curation; funding acquisition; writing – review and editing. Lanan Li: conceptualization; methodology; formal analysis; data curation; funding acquisition; writing – review and editing. All authors have read and agreed to the published version of the manuscript.

## Ethics Statement

Animal handling and treatment strategies including experiments were carried out according to the standard guidelines of the Ethics Committee of Lanzhou University Second Hospital. Animal usage protocols were approved by the Ethics Committee of Lanzhou University Second Hospital (D2024‐936).

## Conflicts of Interest

The authors declare no conflicts of interest.

## Supporting information


**Figure S1.** SAB treatment alleviates injury of renal tubular epithelial cells in FAinduced AKI mice. (A) The schematic diagram of experimental design. (B) Representative gross‐morphological images of kidney cross section. (C–E) The creatinine, BUN and ALT/AST levels (*n* = 6). (F, G) HE staining of kidney sections and tubuler injure score. (H‐I) Masson staining of kidney sections and statistical analysis. (J‐K) WB analysis of KIM‐1. (L‐M) Immunohistochemical analysis of KIM1. *p < 0.05; **p < 0.01; ***p < 0.001.

**Figure S2.** SAB treatment alleviates FA‐induced renal inflammatory response in mice. (A–C) qRT‐PCR analysis of TNF‐α, IL‐1β and MCP‐1. (D–G) Immunohistochemical analysis of TNF‐α and IL‐1β. (H, J) WB analysis of TNF‐α and IL‐1β. *p < 0.05; **p < 0.01; ***p < 0.001, ns, not significant.

**Figure S3.** SAB inhibits FA‐induced lipid peroxidation and ferroptosis in AKI mice. (A‐D) qRT‐PCR analysis of GPX4, ACSL4, SLC7A11 and COX‐2. (E‐F) Immunohistochemical analysis of 4‐HNE. (G–K) WB analysis of GPX4, SLC7A11 FSP1 and PRDX5. (L) Detection of MDA levels. (M) Detection of GSH levels. *p < 0.05; **p < 0.01; ***p < 0.001.

## Data Availability

The data that support the finding of this study are available from the corresponding author upon reasonable request.
