# Supplementary figures and images for: Salvianolic Acid B Attenuates Ferroptosis in Acute Kidney Injury by Targeting PRDX5
Source: FASEB J. 2025 Jul 14;39(14):e70803. doi: 10.1096/fj.202500258RR (PMC12257431; doi:10.1096/fj.202500258RR)

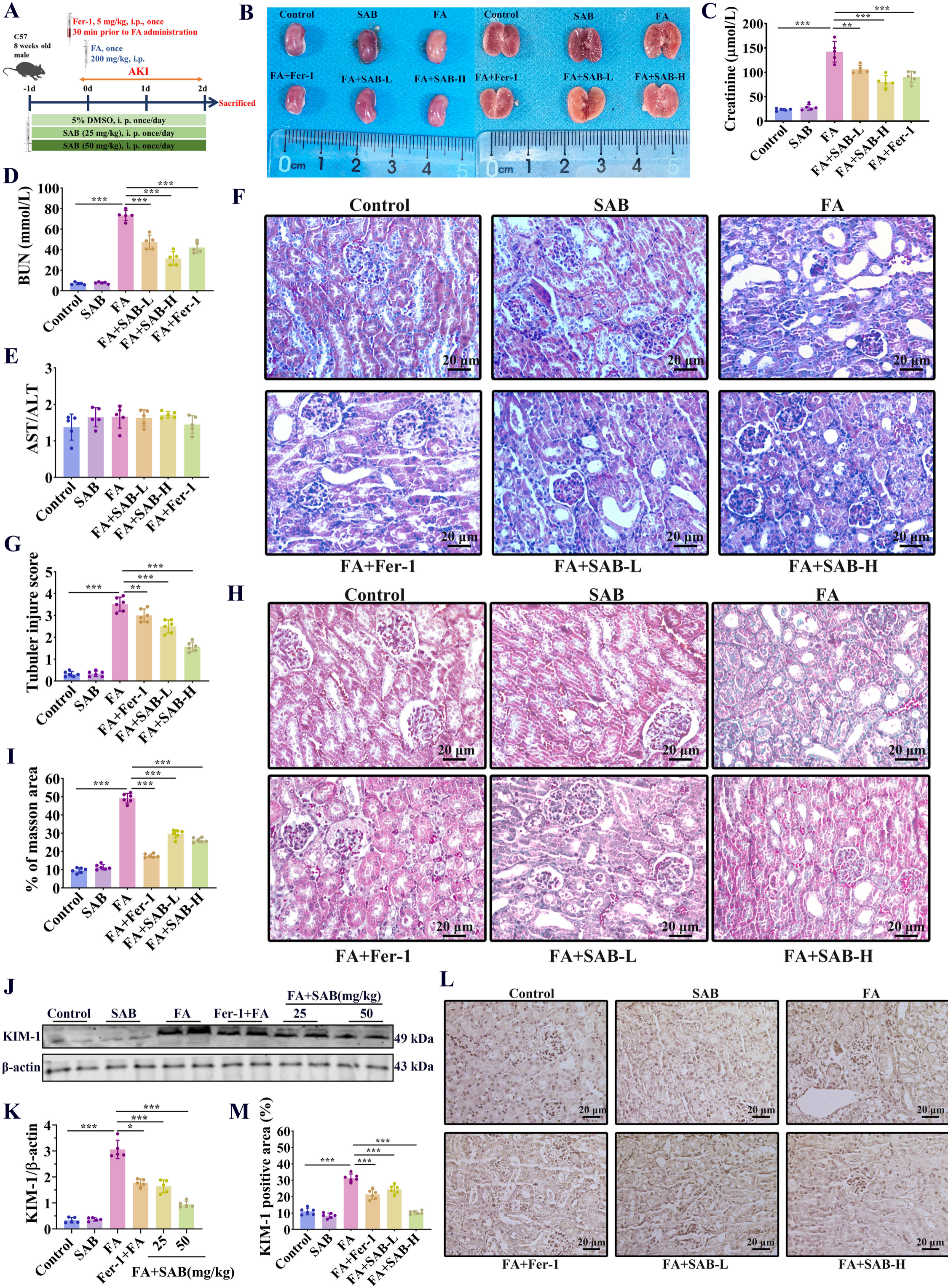

Supplement: Supplementary file 1 — Figure S1. SAB treatment alleviates injury of renal tubular epithelial cells in FAinduced AKI mice. (A) The schematic diagram of experimental design. (B) Representative gross‐morphological images of kidney cross section. (C–E) The creatinine, BUN and ALT/AST levels (n = 6). (F, G) HE staining of kidney sections and tubuler injure score. (H‐I) Masson staining of kidney sections and statistical analysis. (J‐K) WB analysis of KIM‐1. (L‐M) Immunohistochemical analysis of KIM1. *p < 0.05; **p < 0.01; ***p < 0.001. [file FSB2-39-e70803-s001.pdf]

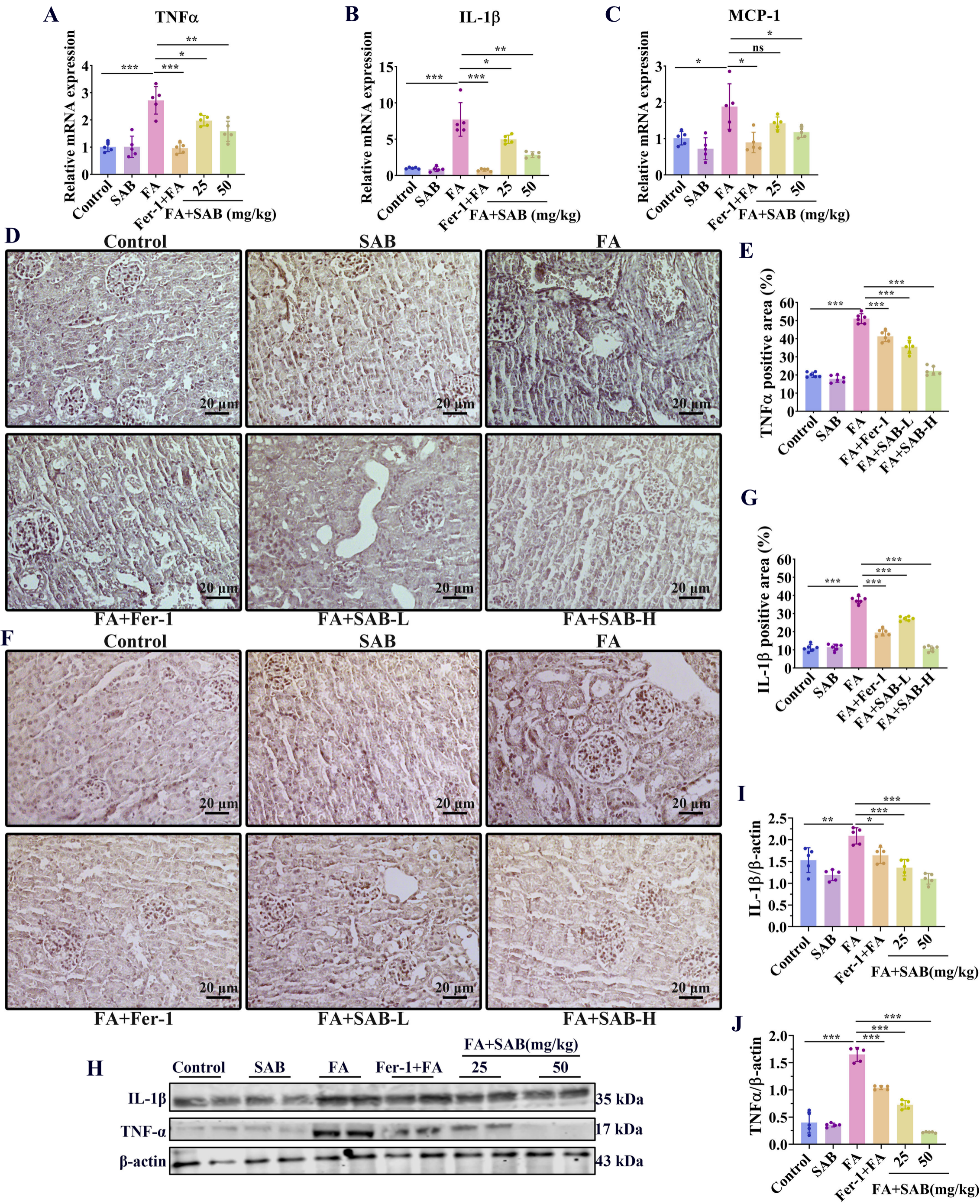

Supplement: Supplementary file 2 — Figure S2. SAB treatment alleviates FA‐induced renal inflammatory response in mice. (A–C) qRT‐PCR analysis of TNF‐α, IL‐1β and MCP‐1. (D–G) Immunohistochemical analysis of TNF‐α and IL‐1β. (H, J) WB analysis of TNF‐α and IL‐1β. *p < 0.05; **p < 0.01; ***p < 0.001, ns, not significant. [file FSB2-39-e70803-s003.pdf]

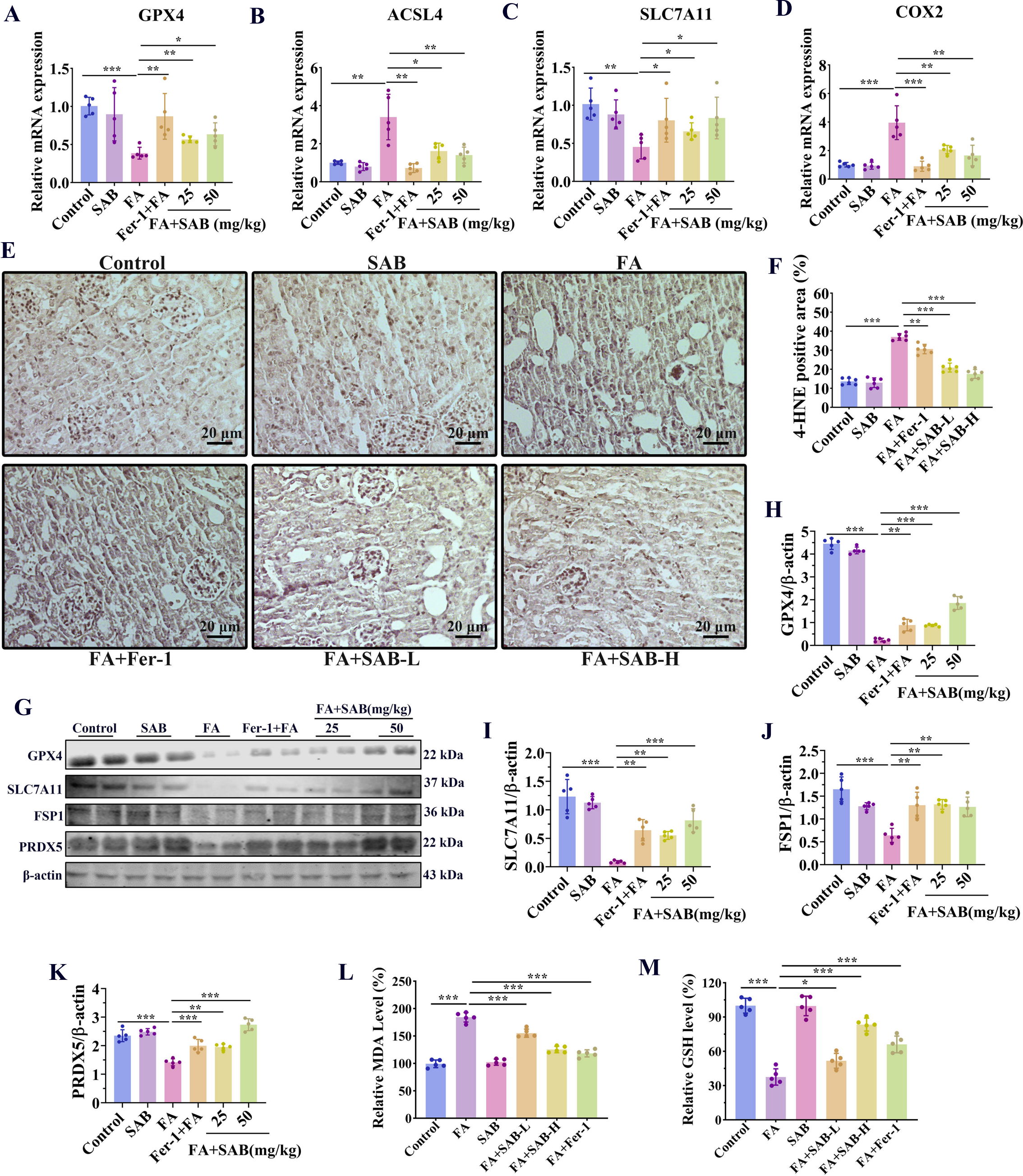

Supplement: Supplementary file 3 — Figure S3. SAB inhibits FA‐induced lipid peroxidation and ferroptosis in AKI mice. (A‐D) qRT‐PCR analysis of GPX4, ACSL4, SLC7A11 and COX‐2. (E‐F) Immunohistochemical analysis of 4‐HNE. (G–K) WB analysis of GPX4, SLC7A11 FSP1 and PRDX5. (L) Detection of MDA levels. (M) Detection of GSH levels. *p < 0.05; **p < 0.01; ***p < 0.001. [file FSB2-39-e70803-s002.pdf]
